# Engineering Polymeric Nanosystems against Oral Diseases

**DOI:** 10.3390/molecules26082229

**Published:** 2021-04-13

**Authors:** Valeria Mercadante, Edoardo Scarpa, Valeria De Matteis, Loris Rizzello, Alessandro Poma

**Affiliations:** 1 Division of Oral Medicine, UCL Eastman Dental Institute, Bloomsbury Campus, Rockefeller Building, 21 University Street, London WC1E 6DE, UK; v.mercadante@ucl.ac.uk; 2 Department of Pharmaceutical Sciences (DISFARM), National Institute of Molecular Genetics (INGM), Via G. Balzaretti 9, 20133 Milan, Italy; edoardo.scarpa@unimi.it (E.S.); loris.rizzello@unimi.it (L.R.); 3 National Institute of Molecular Genetics (INGM), Via F. Sforza 35, 20122 Milan, Italy; 4 Department of Mathematics and Physics “Ennio De Giorgi”, Via Monteroni, c/o Campus Ecotekne, 73100 Lecce, Italy; valeria.dematteis@unisalento.it; 5 Division of Biomaterials and Tissue Engineering, UCL Eastman Dental Institute, Royal Free Hospital, UCL Medical School, Rowland Hill Street, London NW3 2PF, UK

**Keywords:** nanotechnology, polymeric nanoparticles, oral diseases, oral medicine, caries, restorative dentistry, endodontology, periodontology, oral cancer, pre-clinical models, clinical studies

## Abstract

Nanotechnology and nanoparticles (NPs) are at the forefront of modern research, particularly in the case of healthcare therapeutic applications. Polymeric NPs, specifically, hold high promise for these purposes, including towards oral diseases. Careful optimisation of the production of polymeric NPs, however, is required to generate a product which can be easily translated from a laboratory environment to the actual clinical usage. Indeed, considerations such as biocompatibility, biodistribution, and biodegradability are paramount. Moreover, a pre-clinical assessment in adequate in vitro, ex vivo or in vivo model is also required. Last but not least, considerations for the scale-up are also important, together with an appropriate clinical testing pathway. This review aims to eviscerate the above topics, sourcing at examples from the recent literature to put in context the current most burdening oral diseases and the most promising polymeric NPs which would be suitable against them.

## 1. Introduction

Oral health is an integral element of overall health and wellbeing, enabling essential daily functions such as eating, speaking, smiling, and socialising [1]. Oral diseases include a range of chronic clinical conditions that affect the soft and hard tissues of the mouth, including dental caries (tooth decay), periodontal (gum) disease, and oral cancers, which can have a significant impact on function, aesthetics, and quality of life [2].

Despite being largely preventable, oral diseases are a major global public health problem, with the Global Burden of Disease Study 2017 [3] estimating that oral diseases affect close to 3.5 billion people worldwide, predominantly untreated dental caries in the deciduous and permanent dentitions, severe periodontal disease, and edentulism. Furthermore, lip and oral cavity cancers were among the top 15 most common cancers in the world in 2018 [4].

Dental caries begins with the localised destruction of dental hard tissues (enamel and dentine) caused by the bacterial fermentation of free sugars [5]. While if recognised during the early stages it can be arrested and possibly reversed especially with exposure to fluoride, once the caries progresses and spreads to the dental pulp it can cause infection and ultimately, if untreated, sepsis and tooth loss [6].

Periodontal diseases are chronic inflammatory conditions affecting the tissues surrounding and supporting the teeth. The initial reversible inflammation of the periodontal soft tissues is called gingivitis and presents clinically with gingival bleeding and swelling. In susceptible individuals a progressive destruction of the periodontal tissue support, called periodontitis, can occur and lead to tooth loss [7].

Cancer of the oral cavity accounts for approximately 3% of all malignancies and has a 5-year survival rate of 50–60% [8]. Current management includes the use of radiation, surgery, and chemotherapy with potentially significant long-term side effects. Many oral squamous cell carcinomas (OSCC), which are the most common type of oral cancers, develop from potentially malignant disorders, such as leukoplakia, erythroplakia, oral lichen planus [9,10], and oral submucous fibrosis [11].

Nanomaterials and NPs are currently extremely attractive materials to be investigated for a plethora of applications, ranging from sensing/diagnostics to drug delivery and therapy. The final architecture and composition of the nanomaterial to be produced is normally strictly dependent on the ultimate usage. The popularity of NPs originates from the intriguing characteristics deriving precisely from the small scale, which include an extremely high-surface area to volume, but coupled with synthetic versatility which results in adjustable surface charge, tailorable cargo-loading and cargo-release capability, and ultimately versatility for the surface decoration with specific chemical moieties. This latter feature, together with the type of material (e.g., polymers) used for the fabrication, can guarantee extremely high biocompatibility/biodegradability, paramount in the case of healthcare and drug-delivery applications in medicine and dentistry [12,13,14].

Indeed, nanotechnology has been studied in dentistry since 1970 to promote oral health [15]. It has been evaluated to manufacture composites with reduced shrinkage and improved wear resistance, to treat dental hypersensitivity using gold NPs in occluding dental tubules, for tooth repair by stimulating dentin formation and pulp regeneration, to prevent caries by destroying pathogenic bacteria residing in the plaque [16]. NPs capable of achieving high local bioactivity, as well as slowly releasing antibacterial agents have been developed and recently exploited for periodontal regeneration [17,18], dental renaturalisation, local anesthesia, treatment of dentin hypersensitivity, and even bleaching [19,20,21,22]. With regards to the oral soft tissues, nanotechnology has been investigated to improve the screening and detection of oral cancer and the local administration of drugs for several oral diseases [23].

Taking into consideration that the use of nanotechnology and NPs has the potential to have a major impact on the prevention, diagnosis, and treatment of oral diseases, the rationale at the base of this review is to bring together scientists and clinicians to provide an expert perspective on the current trends and challenges related to the “bench-to-bedside” process of polymeric NPs, aiming at highlighting promising research avenues and translational strategies to successfully develop polymeric NPs for oral diseases.

## 2. NPs for Oral Diseases: Types of Polymers and Synthetic Methods

**Figure 1:**
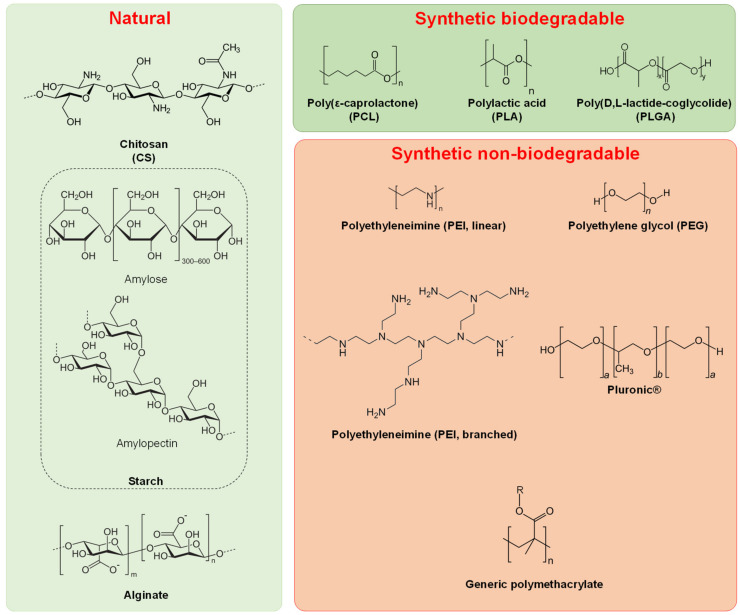
Chemical structures of the main polymers used to produce nano particles (NPs) against oral diseases. These are grouped in natural polymers, as well as biodegradable and non-biodegradable synthetic polymers.

Bearing in mind the final therapeutic goal, the polymeric materials used to generate NPs aimed at managing oral diseases have to exhibit physico-chemical stability, coupled with high biocompatibility, and possibly biodegradability (depending on the ultimate usage). Different types of polymers have been used for the production of NPs, ranging from purely natural to semi-synthetic to fully synthetic or mixtures [24] (Figure 1).

One of the most important natural polymers extensively investigated for the production of this type of NPs is chitosan (CS, poly(1,4)-*b*-d-glucopyranosamine) [24,25]. CS is a natural hydrophilic polycationic polysaccharide, derived from alkaline deacetylation of chitin, the second most abundant natural biopolymer which is the principal component of crustacean exoskeletons [26,27]. CS has received significant interest in the fields of biomedicine, food industries, agriculture, and environmental science [28]. This hydrophilic biopolymer offers an attractive material for chemical modifications and grafting reactions with other reactive molecules due to the free amine and hydroxyl groups [29]. These very same chemical functionalities allow it to bind effectively to negatively charged substances via electrostatic interactions or hydrogen bonding, hence CS is considered extremely useful as a vehicle for mucoadhesive drug delivery and is also able to accelerate wound healing and alleviate pain [30,31]. Furthermore, CS exhibits a broad range of antimicrobial activity (against both bacteria and fungi) [27,32] depending on the molecular weight (MW) and the degree of deacetylation [24,33,34], and has biocompatible and biodegradable properties [29,33]. These latter, together with the above-mentioned properties render it one of the most tested polymers for drug delivery (included in the European Pharmacopeia since 2002) [35,36], and currently commercially employed as a sustained drug release matrix [25,29,36,37,38].

CS can also be modified to introduce specific functional groups for apt purposes, e.g., to improve mucoadhesiveness or alter solubility. In this respect, thiol-modified CS (TCS) is extremely popular, prepared via the derivatisation of the primary amino groups of CS with coupling reagents bearing thiol functions. To date, three different TCS derivatives have been synthesised: CS-thioglycolic acid conjugates, CS-cysteine conjugates, and CS-4-thio-butyl-amidine (CS-TBA) conjugates. These TCS have numerous advantageous features in comparison to unmodified CS, such as significantly improved mucoadhesive and permeation enhancing properties. The strong cohesive properties of TCS make them highly suitable excipients for controlled drug release dosage forms [36,39]. With the aim of increasing CS solubility in neutral and basic solutions without affecting other important characteristics, carboxymethyl CS is prepared by carboxymethylation of the hydroxyl and/or amine moieties of CS [40,41].

CS is also actively exploited to produce mixtures with other materials [e.g., polymers such as poly(ε-caprolactone) (PCL) or poly(d,l-lactide-*co*-glycolide) (PLGA), or even clay] to render them mucoadhesive or improve their control on drug release, *de facto* improving their biophysical, and/or pharmaceutical properties [42,43,44]. Clay-polymer nanocomposites are getting to play a role in nanoformulations for drug delivery, thanks to their strong effect on controlled drug release, while clay provides excellent sorption properties due to its rough and porous surface, and it is cost-effective, hydrophilic, and nature friendly [44].

Other natural polymers include starch, an inexpensive hydrophilic polysaccharide that is gaining great attention due to its biodegradability, biocompatibility, non-toxicity [45], and alginate. This latter is an anionic water-soluble polysaccharide which is also biocompatible, biodegradable, and able to produce gels with polyvalent cations. It is used in controlled drug delivery systems, particularly for the oral route [46,47].

Amongst synthetic polymers, PCL and PLGA are extensively studied for the production of nanomaterials aimed at treating oral diseases. PCL possesses several advantages that include: biocompatibility, biodegradability, high encapsulation capacity, non-toxicity, absence of generation of an acidic environment during degradation (in contrast with PLGA and polylactic acid, PLA), and slower degradation than PLGA, which makes it more suitable for controlled drug delivery [48,49,50]. Even surface-coating with gangliosides has been reported [51].

PLGA has been widely explored to produce drug delivery systems and tissue engineering scaffolds. It possesses many well-described features: excellent biocompatibility, biodegradability, and controllable release kinetics, in addition to extensive applications in the clinic [52,53,54,55]. The most widely used synthetic polymeric NPs to date are composed of PLGA. The physicochemical properties of PLGA, biodegradation rate, and in vivo behaviour can be modified by manipulating MW, lactic acid:glycolic acid ratio, and end group [56,57]. The biodegradable property of PLGA is due to the products of its hydrolysis, lactic acid, and glycolic acid [57,58]. Since these two compounds are endogenous and easily metabolised by the body via the Krebs cycle, a minimal systemic toxicity is associated with the use of PLGA for drug delivery or biomaterial applications [56]. For these reasons, PLGA is approved by the US Food and Drug Administration (FDA) and European Medicine Agency (EMA) in various drug delivery systems in humans [59]. However, the acidic by-products released from PLGA could decrease the pH in the surrounding tissues and activate endogenous proteases, which is considered a critical concern for certain potential applications, that requires further investigation [57,60].

A peculiar backbone-degradable pH-responsive polymer, hyperbranched polyacylhydrazone (HPAH), was successfully synthesised by polycondensation of diketone and trihydrazine [61]. HPAH possesses a large number of acylhydrazine terminals for further conjugation of drugs and exhibits good water solubility and low cytotoxicity, making it an excellent carrier for drug delivery. HPAH combines high stability and stimuli-responsiveness together, and it can be degraded into low-MW products due to the existence of acid-sensitive acylhydrazone bonds in the backbone. These properties render HPAH a promising biomaterial for biomedical applications, particularly to carry hydrophobic drugs [62].

Amongst synthetic polymers, polyethylene glycol (PEG) block-copolymers have also been extensively used. PEG confers stealth properties to the formulation, which allow the NPs preparation to be less avidly taken up by the reticuloendothelial system and retained in the circulation for a longer period of time [63,64,65]. Many PEG-based block copolymers are well studied and exhibit an extremely satisfactory safety profile (e.g., FDA-approved Pluronics^®^) [66,67]. PEG-PLGA, PEG-PCL, and PEG-PLA (normally synthesised via ring-opening polymerisation using PEG as a macroinitiator in the presence of an adequate catalyst, such as stannous octanoate) exhibit excellent systemic characteristics and biodegradability [64,68,69] and can be easily derivatised with suitable moieties (e.g., peptides) to achieve active targeting [70,71,72,73] or even with drugs to achieve sustained drug release [74,75].

The above-mentioned copolymers, though, are neutral, therefore their mucoadhesion capability is extremely poor. Amongst synthetic polycations, polyethyleneimine (PEI) is a linear or branched polymer containing primary, secondary, and/or tertiary amines. It possesses low cytotoxicity (no sign of acute or chronic toxicity of PEI solution has been reported in rats and rabbits when given orally or subcutaneously) [76,77], high transfection efficiency, and pH buffering capacity. Indeed, PEI is often described as a “proton sponge” since its charge density is dramatically pH dependent [76,78]. PEI is commonly applied for drug delivery, coating magnetic NPs or as a transfection agent [76,79,80,81]. Mucoadhesive NPs made of oppositely charged polymers PEI and dextran sulfate (DS) have been reported, since PEI and DS are hydrophilic macromolecules containing numerous hydrogen bond-forming groups which have been suggested as being adhesive to mucous membranes, particularly those within the oral cavity [76,80].

In comparison to PEI, PEG-PEI block-copolymers possess superior biocompatibility, increased transfection efficiency and polymer solubility, prolonged circulation time, and decreased nonspecific interaction with serum proteins [79,80].

Other polymers widely used to prepare NPs for oral health are polymethacrylates, usually synthesised via free radical polymerisation or more controlled approaches such as atom transfer radical polymerisation (ATRP) and reversible addition-fragmentation chain transfer (RAFT) polymerisation. These types of syntheses provide the highest flexibility in terms of chemical and physicochemical properties and architecture for the final polymer. In particular, ATRP and RAFT provide precise control over polymer MW and polydispersity indexes (PDI < 1.3). Residues bearing cationic, neutral or anionic groups can be easily introduced (e.g., methacrylic acid or dimethylaminoethyl methacrylate) [17,21,82,83,84,85,86,87,88,89,90], and even commercial methacrylate-based products (Eudragit^®^) have been used in the preparation of NPs for oral health applications [90,91,92,93,94,95,96,97,98,99]. There are significant advantages in using these materials (e.g., increased solubilisation of drugs, stimuli-responsiveness, etc.), but a major drawback is related to their lack of biodegradability, which could significantly hinder the final regulatory approval of the designed NPs depending on the envisaged application.

## 3. NPs for Oral Diseases: Production and Drug Loading Methods

A plethora of methods have been used to generate NPs for oral health applications, with each of these methods exhibiting its own advantages and disadvantages [100,101]. However, probably the main factor influencing the choice is related to the polymeric materials used and the drug/molecule to be loaded into the NPs.

A general method relies on carrying out a cross-linking reaction onto the main polymer. This cross-linking can take place via the establishment of chemical bonds or through strong physical interactions. Chemical cross-linkers possess two reactive functional groups, therefore allowing the formation of covalent bonds between polymeric chains. Various cross-linkers are available, and their applicability is dependent from the functional moieties available on the polymer. For example, if the polymer bears amino groups, then aldehydes, epoxides, and cyanates can be used [34,35].

Apart from chemical cross-linking, physical cross-linking has also been reported. One of the simplest and mildest procedures is based on ionic gelation involving electrostatic complexation between the polymer and an oppositely charged species, such as polyanionic sodium triphosphate (TPP). This latter in particular has been widely explored for fabricating CS NPs [34,102,103]. Due to the cross-linking effect when the two aqueous solutions are mixed, the polymer precipitates and forms NPs. The characteristics of the structures prepared by this method are dependent on several formulation variables, including polymer MW and concentration, and the cross-linker-to-polymer ratio. Recent studies have reported that more compact and smaller NPs with narrower size distributions were produced in the presence of moderate amounts of salt due to the salt-induced screening effect [100,103,104,105]. This shows in fact that the ionic strength of the solvent is also an important parameter to address in the preparation of polymeric NPs by ionic gelation.

A similar mild approach does not involve specific cross-linking mediated by multivalent ions, but the electrostatic interaction between oppositely charged polymers (e.g., PEI and DS) under aqueous conditions. This technique is called polyelectrolyte complexation, and the ionic interaction between the oppositely charged polymers leads to the formation of self-assembled NPs at room temperature [76,106].

Other methods include nanoprecipitation [22,48,49,89,91,92,93,95,96,97,98,99,101,107,108], emulsification diffusion [109,110], and emulsification-evaporation methods [36,52,90].

Nanoprecipitation, also known as the solvent displacement method, employs the use of a water-miscible organic solvent in which the polymer (or polymers) intended to prepare NPs is dissolved. In general, the drug is also dissolved in the organic solvent based on its solubility. The method is based on spontaneous miscibility of the organic solvent and its subsequent diffusion into the aqueous phase (in the presence or absence of a suitable stabiliser or surfactant) leading to the precipitation or self-assembly process of the polymer, therefore resulting in NPs formation [22,48,49,89,91,92,93,95,96,97,98,99,101,107,108]. Even in presence of the same polymer (e.g., PLGA), the usage of different methods can exert a dramatic effect on the physico-chemical properties of the resulting NPs [59].

The emulsification diffusion [109,110] and emulsification-evaporation methods [36,52,90] consist of the preparation of a conventional oil-in-water emulsion under mechanical stirring or phase inversion. The internal organic phase includes the drug and the polymer, whilst the external aqueous phase consists of one or several hydrophilic stabilisers. Once the emulsion is obtained, in the first method it is diluted with a sufficient amount of water to dissolve the inner organic phase, whilst in the second method a rapid displacement of the solvent from the internal into the external phase is achieved by evaporation under reduced pressure [111].

All the above-mentioned approaches can also be used in microfluidic reactors, therefore standardising more effectively the production conditions [66].

Depending on the polymer synthesis process, in some cases the production of the NPs can be performed simultaneously to the polymerisation reaction. For example, this is the case for precipitation polymerisation reactions, where the monomers are soluble into the reaction solvent, but the polymer is not, therefore precipitating into the non-solvent medium as NPs [17,21,83,84,85,86,87,88,112,113].

In terms of loading of the active payload (e.g., drugs, DNA/RNA or proteins) into the NPs, this can be either dispersed/embedded/encapsulated into the polymer matrix or alternatively it can be adsorbed onto the surface of NPs [13,35,114,115,116]. In this case, though, the interaction between NPs and bioactives might be relatively weak, therefore potentially causing issues with the overall stability of the designed NPs (e.g., upon storage) as well as potential burst-release effects when administered [13,117]. An important advantage of the encapsulation/loading process is related to the potential improvement of undesired properties of the bioactive itself. As an example, encapsulated photosensitisers can be quenched, resulting in loss of phototoxicity [118,119]. Similarly, the envelopment of inorganic NPs using biocompatible polymers further lowers the toxicity [38,120,121]. In this latter case, however, intermediate complexing agents might be necessary to improve the loading capacity of the polymer matrix [e.g., in the case of Cu(II) and CS] [38].

## 4. Applications of Polymeric NPs for Oral Diseases

### 4.1. Caries and Hypersensitivity

Dental caries, also known as tooth decay, is a biofilm-dependent infectious disease and is one of the most common bacterial infections in humans [5].

The primary mineral component of the hard dental tissues (enamel and dentin) and target for the bacterial acids is hydroxyapatite [Ca_5_(PO_4_)_3_(OH)]. At physiological conditions, the oral fluids (saliva, biofilm fluid) have calcium (Ca^2+^) and phosphate (PO_4_^3−^) in supersaturated concentrations, and these ions are continually deposited on the enamel surface [122]. With the presence of bacterial acids, the pH decreases with the chemical dissolution of calcium and phosphate, and therefore mineral loss (demineralisation) when the pH remains below ~6.5 for dentin and ~5.5 for the enamel [123]. The acidic microenvironment indeed allows for fermentable carbohydrate to be consumed by the cariogenic bacteria, with the *S. mutans* being the major caries-related species [124].

Once bacterial acids have caused tooth decay, the treatment involves removing the carious tissues and filling the tooth cavity with a restorative material with good esthetic and performance, such as a composite [125]. The composite restoration is bonded to the tooth structure via an adhesive but its longevity is often compromised by caries at the restoration margins [126]. It would be therefore highly desirable for the composite and bonding agent to possess antibacterial and remineralisation capabilities.

Nanotechnology could provide novel strategies in the prevention and treatment of dental caries, specifically in the control and management of dental plaque biofilms and remineralisation [127].

The group of Toledano extensively studied PolymP-nActive NPs (nanoMyP) of ~100 nm in diameter made of 2-hydroxyethyl methacrylate as a backbone monomer, ethylene glycol dimethacrylate as a cross-linker, and methacrylic acid as a functional monomer. The different functionalities allowed loading a number of ions and antibiotics for antibacterial and regeneration purposes. One of the first works in this area was related to the preparation of zinc-loaded NPs into a dental adhesive system to facilitate the inhibition of collagen degradation mediated by matrix metalloproteinases (MMPs), and to provide calcium ions for mineral deposition within the resin-dentin bonded interface. Although NPs failed to infiltrate demineralised intertubular dentin and remained on top of the hybrid layer, they did not alter the bond strength, and were covered by calcium and phosphorus within 24 h. Furthermore, NPs application in etched dentin reduced MMP-mediated collagen degradation [18]. NPs loaded with calcium, zinc, and doxycycline (Dox) exhibited a sustained release over 21 days, with the highest antibacterial effect exhibited by the Dox NPs (60 to 99% reduction of planktonic bacteria) followed by Ca NPs or Zn NPs (30 to 70% reduction). *P. gingivalis, S. mutans,* and *L. lactis* were the most susceptible bacteria, whilst *S. gordonii* and *S. sobrinus* exhibited the highest resistance to the tested NPs [87]. Zn and Dox NPs were subsequently tested to contrast the formation of cariogenic biofilms on dentin disks (*vide infra*). Results highlighted the highest bacterial decrease rate using Zn NPs (87%), followed by Dox NPs (32%). In addition, Zn NPs exhibited the highest mineralisation degree. Although Dox NPs had an effect on the mechanical properties due to a promotion of the collagen cross-linking, their application for remineralisation was not advised by the authors, who also pointed out that a potential dysbiosis phenomena with commensal bacteria might take place when using these types of NPs, and should therefore be assessed before clinical translation. Furthermore, adequate in vivo testing should be carried out [88].

Aiming at a potential treatment for caries and dentin hypersensitivity, the same authors evidenced that crystalline and amorphous phases of newly formed hydroxyapatite were observed in dentine treated with Zn NPs as well as Ca NPs, with multiple shapes of crystallites. Zn NPs, though, resulted in a higher level of crystallinity. Both Zn NPs and Ca NPs exhibited a reduction in dentinal fluid flow, but in this case Ca NPs acted faster (100% occlusion within 24 h). Dox NPs were also tested (since Dox should promote osteogenic differentiation), but the levels of crystallinity and flow reduction achieved were remarkably low [21,84,86]. Further testing should evaluate the long-term effect of NPs (e.g., at 3 or 6 months), as well as resistance to environmental acids under circumstances similar to oral conditions. NPs loaded with silver were also tested (aiming at potentially enhanced antimicrobial properties), but were not successful in exhibiting remineralisation properties [83].

With the purpose of elucidating the anticariogenic effects of CS and CS NPs, especially against oral streptococci, Aliasghari et al. [128] assessed the antibacterial and adhesion properties of these materials for *S. mutans*, *S. sobrinus*, *S. sanguis,* and *S. salivarius*. Although both CS and CS NPs exhibited antimicrobial activity, the average dosage required for CS NPs was lower than CS to achieve the same effect. Furthermore, both CS and CS NPs reduced biofilm formation, particularly of *S. mutans* (up to ~93%). It would have been interesting to test these properties in vivo and compare them with existing commercial products (e.g., mouthwash) to further confirm the antimicrobial properties of these materials.

Aiming to attenuate the acid tolerance typical of microflora responsible of caries development, Neilands et al. [24] assessed the acid tolerance of *S. mutans* with or without the presence of CS NPs in mildly acidic pH. The subsequent exposure to low pH and viability evaluation confirmed that CS NPs were indeed able to hinder the acid tolerance response in bacteria. These are extremely promising results, although still preliminary. It would be interesting to confirm the mechanism for this effect mediated by the CS NPs, as well as verify whether this is limited to *S. mutans* or would be translatable to complex biofilm systems.

**Figure 2:**
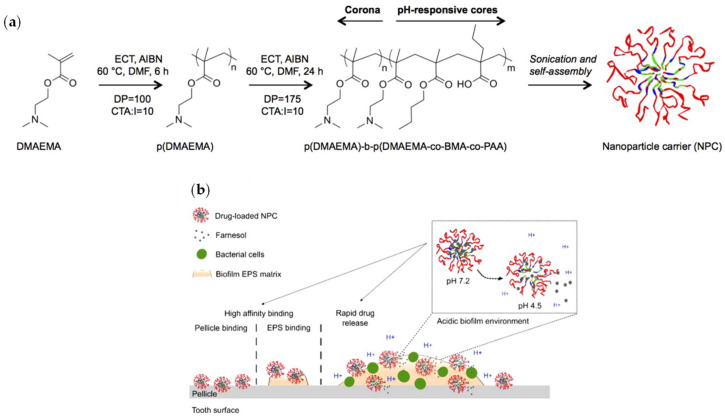
(**a**) Illustration of the chemical synthesis and self-assembly of the di-block copolymers used in the work of Horev et al. (**b**) Proposed mode of action of pH-responsive NPs for prevention and/or treatment of biofilms. PDI: polydispersity index; ECT: chain transfer agent (CTA), 4-cyano-4-[(ethylsulfanylthiocarbonyl)sulfanyl]pentanoic acid; AIBN: initiator, 2,2-azobisisobutyronitrile; DMF: dimethylformamide; DP: degree of polymerisation. Adapted with permission from Horev et al. [89].

To avoid rapid clearance from biofilm-tooth interfaces, Horev et al. [89] developed polymethacrylate pH-responsive micellar NPs able to bind to bare, as well as saliva-coated hydroxyapatite and exopolysaccharide matrixes (Figure 2).

The obtained micelles were cationic, 20 nm in diameter, and were loaded with farnesol (a strong antibacterial), with a drug loading of >20% wt. The micelles were tested using a system that mimics the oral environment clearance due to the saliva elimination. The drug release was enhanced in acidic conditions (pH 4.5), typical of the bacteria environment. This permitted achieving the reduction of carious diseases onset with a disruption (even mechanical) of *S. mutans* biofilms 4-fold more effective than free drug in vivo in a rodent dental caries model.

Nguyen et al. [100] created a CS NPs formulation to improve the local delivery of fluoride. When present in sub-ppm concentration during an acidic attack (as it is for invasion by cariogenic bacteria), fluoride is adsorbed onto the surface of the apatite crystals where it inhibits tooth demineralisation, promotes regeneration, and functions as an antimicrobial. CS NPs of ~100 nm in diameter were prepared via ionic gelation in the presence of NaF. Although the entrapment efficiency was relatively low (3.6 to 6.2%), the authors showed a promising release profile in conditions simulating the acidic attack in vitro over 24 h and concluded that such formulation could be beneficial for protecting against caries development. It would have been interesting to assess this effect also on ex vivo or even in vivo caries models, especially in comparison with conventional NaF administration.

Many groups have assessed the possibility of vehiculating flavonoid compounds as anticaries using NPs. Patil and Jobanputra confirmed that rutin (a flavonoid extracted from *Ruta graveolens*) can indeed be entrapped into CS NPs and its bactericidal and bacteriostatic effect on cariogenic bacteria enhanced, together with the improvement of its physico-chemical stability in simulated saliva (although for a limited timespan). It would have been interesting to assess the performance of these NPs in a more complex caries model, possibly in vivo, and also assess the biocompatibility of these drug delivery systems in the oral biological environment [35].

Tiyaboonchai et al. developed mucoadhesive PEI-DS NPs loaded with *Punica granatum* peel extract (rich in polyphenolic compounds). A high drug entrapment efficiency was observed (~98%), although the mass ratio of PEI:DS significantly affected both particle size and entrapment efficacy. The mucoadhesiveness, drug release, and antibacterial properties of the NPs, though, could be improved. Indeed, ~25% of NPs were left after 2 h of ex vivo wash-off testing on porcine buccal mucosa, and the drug was completely released in vitro in 3 h. In addition, usage of the NPs did not allow to reduce the dosage of the drug for antibacterial purposes, which would be expected when recurring to NPs [76].

A pH responsive, mucoadhesive protein-polysaccharide (sodium caseinate-sodium alginate) coacervate was used to synthesise nisin-loaded NPs to prevent and disrupt oral biofilms, specifically of *E faecium*, *S. epidermidis,* and *E. faecalis*. The NPs formed at pH 5 exhibited the highest nisin encapsulation efficiency (~75%), with ~240 nm diameter and negative zeta potential, although their polydispersity was quite high. NPs efficiently controlled the bacterial growth, inhibiting as well as eradicating pre-formed oral biofilms more efficiently than the free nisin, without exhibiting evident cytotoxic effects (although this was not tested on oral cell types). Ex vivo studies confirmed the mucoadhesive properties, but the drug was completely released within 30 min from the application, therefore highlighting that more optimisation is required towards the clinical translation of these NPs [129].

A similar mucoadhesive platform has been developed by Chronopolou et al., who investigated the production and application of CS, TCS, and CS-PLGA NPs loaded with chlorhexidine (CHX). A sustained drug release was achieved from all NPs over 50 h, except from CS-PLGA NPs. CS-based NPs adhered on human tooth surfaces in a simulated brushing and rinsing process, although this effect was not systematically quantified. Furthermore, some in vitro toxicity (between 20 and 60%, evaluated on human gingival fibroblasts) was observed, whilst antibacterial properties were not evaluated. A significant optimisation and further characterisation are required to implement these NPs into dental products such as mouth rinse or toothpaste [30].

**Figure 3:**
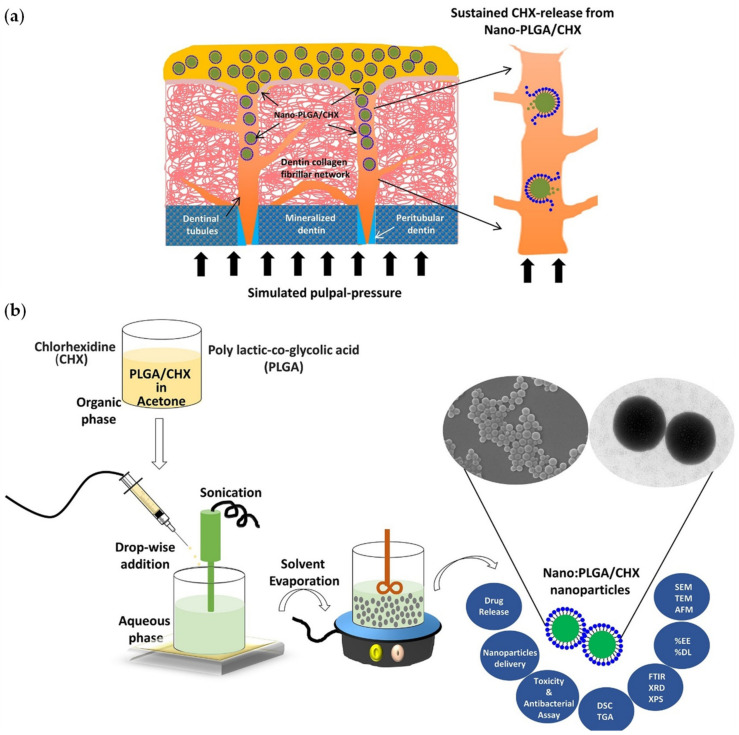
(**a**) Schematic diagram showing the proposed principle of delivering the active drug (CHX) to the demineralised dentin-substrates in the form of CHX-loaded PLGA NPs through the micron-sized dentinal-tubules (DT) under the influence of the simulated pulpal-pressure targeting for prolonged drug-release and retention inside the collagen fibers of demineralised dentin matrix. NPs should act as a repository that slowly degrades to provide a constant source for the release of CHX over predetermined time-intervals. The retained NPs inside the dentinal-tubules will slowly release CHX to be carried by the dentinal-fluids present in the dentinal-tubules under the influence of the positive intra pulpal hydrostatic pressure through the 3D interconnected tubular capillary platform and the water-rich interfibrillar spaces of the dentin-collagen network. (**b**) Illustrative sketch showing the preparation of the CHX-loaded PLGA NPs by the emulsion evaporation procedure and their subsequent characterisation, including representative SEM and TEM micrographs. Adapted with permission from Pryiadarshini et al. [130].

Priyadarshini et al. [130], conversely, focused on the integration with demineralised dentin and adhesive substrates by developing CHX-loaded PCL NPs. CHX loading decreased the size of the NPs and caused a positive shift in ζ-potential. The NPs with the best performance exhibited 240 nm diameter, 85% encapsulation efficiency, and 28% drug loading, with low polydispersity. They exhibited antimicrobial activity *vs E. faecalis* and *S. mutans*, but a significant discrepancy was observed between the drug release in vitro (which was almost complete in 25 d) and ex vivo (~20% in the same timespan). Furthermore, a certain amount of cytotoxicity was observed on dental pulp stem cells 48 to 72 h after incubation. SEM and TEM imaging confirmed the deep penetration and retention of the CHX-loaded NPs inside dentinal tubules, and silver-labelling revealed the close association and even distribution of NPs throughout the resin tag structure. A significant optimisation of this system, though, is required before its translation into the clinic [50]. Shifting from PCL to PLGA allowed improving the biocompatibility of the system, although similar drug release issues were observed (Figure 3).

A similar trend was observed also when PLGA NPs were investigated for the delivery of grapeseed extract (GSE) to demineralised dentin, since GSE should improve its biodegradation resistance by cross-linking collagen type I, inhibiting MMPs and decreasing the rate of dentin demineralisation. NPs having PLGA/GSE of 100:75 (*w*/*w*) showed the highest cumulative GSE release and were associated with the best improvement in biodegradation resistance [57]. The discrepancy observed between the release in vitro and ex vivo, however, should be investigated in depth, and a more detailed quantification of the delivery of the NPs into the dentinal tubules should be performed, perhaps using a radiotracer or a tailored fluorescent label.

Genari et al. [90,94] extensively studied the possibility of including polymethacrylate-based NPs loaded with anti-inflammatory indomethacin and antibacterial triclosan into adhesive resins and primers for enhanced caries treatment and restoration. The incorporation did not affect the resin capacity to polymerise, although it lowered the amount of drug released, in an inversely proportional manner in relation to the amount of NPs incorporated into the adhesive. The antibacterial effect followed the burst release behaviour observed from the NPs in vitro, which however exhibited a good biocompatibility profile even when drug-loaded, with cell viability values >90% for all the dosages tested.

Yazdian et al. [44,46] evaluated the possibility of exploiting naturally derived curcumin as anticaries. Indeed, curcumin hinders the bacterial connection to tooth surface and resultantly decreases biofilm formation, but its potential for clinical therapeutic application has been limited due to its low oral bioavailability, poor aqueous solubility, and rapid degradation. The authors tested several natural polymers to prepare NPs, including starch, alginate, and pure CS, and even a novel bio-nanocomposite composed of carboxymethyl starch (CMS)-CS-montmorillonite (MMT). Particle size and curcumin entrapment efficiency (EE) were highly affected by different formulation variables. The MMT addition, though, enhanced the EE (up to 91% when 5% *w*/*w* MMT was included in the formulation). Optimal formulations exhibited a sustained release of curcumin over 96 h and >90% antibacterial activity on *S. mutans* biofilm dental models, therefore confirming the anticaries potential for these drug delivery systems. Further investigation on complex biofilms, however, should be performed.

**Figure 4:**
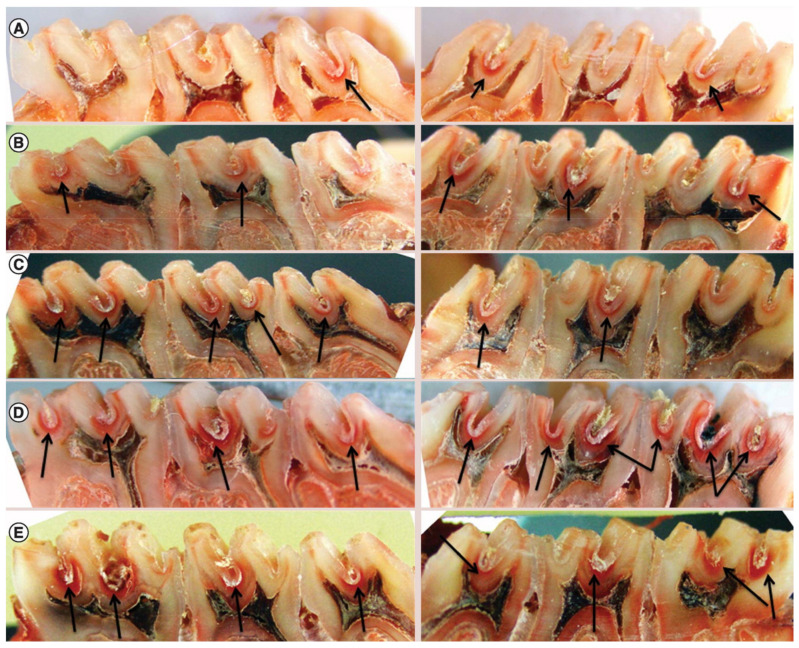
Micrograph showing rat molars after murexide staining and hemisectioning in the mesiodistal sagittal plane. (**A**) The pVAX1-wapA/CSTM NPs intranasal group; (**B**) pVAX1-wapA/CSTM NPs intramuscular group; (**C**) pVAX1wapA intramuscular group; (**D**) pVAX1/CSTM NPs intranasal group; (**E**) pVAX1/CSTM NPs intramuscular group. Arrows indicate the caries lesions. Adapted with permission from Li et al. [131].

An innovative approach has been developed by Li et al., who designed an anticaries DNA vaccine cloning the wapA gene fragment that is involved in *S. mutans* sucrose-dependent adherence and aggregation, and introducing it into the pVAX1 plasmid, which was subsequently loaded onto trimethyl CS NPs. The NPs were tested in vivo in a rat model to evaluate the immune response, which exhibited higher serum IgG and saliva IgA antibody titers in comparison to the controls in the case of intranasal administration. This translated into significantly lower levels of enamel and dentin lesions (Figure 4), potentially paving the way for an immunological-based treatment of caries [131].

### 4.2. Endodontal Disease

The term endodontic treatment covers all aspects of repair and treatment of a tooth in which the pulp has been either damaged or exposed as well as the treatment of periapical tissues [132]. Root canal therapy is performed when the pulp, which is composed of nerves and blood vessels in the tooth, becomes infected or damaged, with the aim of providing a clean root canal and to seal the root at the apex to prevent the ingress of fluids that would otherwise act as a source of nutrients for any bacteria remaining inside the root canal. At the same time, this treatment aims to provide a coronal seal, which prevents the pulp chamber from being re-contaminated by oral flora [132].

Several different types of material have been employed with varying degrees of clinical success in attempts to create a clinically effective seal [133]. The ideal material should be biocompatible, dimensionally stable, able to conform to the individual root canals, and bacteriostatic [132].

Indeed, the complexity of the root canal system renders a complete debridement of bacteria almost impossible, even when conventional methods of endodontic instrumentation and irrigation are performed to the highest technical standards [118].

Nanotechnology could provide novel strategies in the endodontic treatment, and NPs could be incorporated in a sealer, obturating material, intracanal medicament, and irrigating solutions to provide the desired results [134].

With respect to the disinfection of the root canal system, the emerging approach of antimicrobial photodynamic therapy (aPDT) shows good promise. The aPDT is based on the use of a photosensitising agent (a photosensitiser), which can be preferentially localised in certain tissues and subsequently activated by light of the appropriate wavelength in the presence of oxygen to generate cytotoxic reactive oxygen species (ROS) for the target microorganism [108,135]. An advantageous combination of aPDT with NPs, as mentioned above, relies on the fact that the phototoxicity of the photosensitiser agent is quenched when incorporated in the NPs, whilst it is regained when it gets released. Furthermore, other advantages include the fact that NPs include a larger critical mass (concentrated package of photosensitiser) for the production of cytotoxic ROS. Moreover, NPs limit the target cell’s ability to pump the drug molecule back out, thus reducing the possibility of multiple drug-resistance, improving the treatment selectivity and reducing potential immunogenicity risks [118].

Pagonis et al. pioneered the use of PLGA NPs to carry photosensitisers for aPDT. Specifically, they assessed the possibility of using methylene blue (MB)-loaded PLGA NPs in vitro against *E. faecalis*, in planktonic phase and also in experimentally infected root canals of extracted human teeth. The NPs concentrated mainly on the bacterial cell walls, and the synergism of light and MB-loaded NPs led to reductions of 2 and 1 log_10_ CFUs (respectively in planktonic phase and root canals), which were significantly lower than the controls. As correctly highlighted by the authors, however, further studies should define the treatment parameters for optimum endodontic disinfection and the therapeutic window to selectively kill bacterial cells whilst preserving the tissue. Furthermore, a treatment with free MB should have been evaluated amongst the controls [118].

Dasilva et al. extensively evaluated the antibiofilm capability in bovine root segments filled with gutta-percha and sealer incorporated with CS NPs, without and with the canal surface treatment with phosphorylated CS, CS-conjugated rose bengal (CSRB) NPs and aPDT, and a combination of both. The authors found that incorporating CS NPs into the sealer inhibited biofilm formation within the sealer-dentin interface, even after aging for 30 days in comparison to sealers used in their original formulation [136,137]. This effect was maintained when canals were treated with phosphorylated CS [138], and CSRB NPs exhibited penetration into the biofilm structure and disruption of multispecies biofilm with reduction in viability and thickness after aPDT [37], even in the presence of tissue inhibitors (e.g., dentin, dentin-matrix, pulp tissue, bacterial LPS, bovine serum albumin) within root canals [25]. Furthermore, photodynamically activated CSRB NPs caused a significant inactivation of endotoxins and the subsequent reduction of all tested inflammatory markers from activated macrophages [139], whilst incorporation of CS and cross-linking of collagen due to the photodynamic treatment overall increased the resistance of collagen to enzymatic degradation, therefore stabilising dentin [26,29], although some cytotoxicity was observed on murine fibroblasts after aPDT. The same group also confirmed that CS NPs on their own could be used as a final irrigant during the root canal treatment with the dual benefit of removing the smear layer and inhibiting bacterial recolonisation on root dentin [27].

With a more regenerative angle in mind, the same authors prepared CS NPs loaded with dexamethasone with the aim of driving the odontogenic differentiation of stem cells from apical papilla. The loading, release, and biocompatibility characteristics were previously optimised using BSA as a model drug [140]. The authors found that dexamethasone-loaded NPs exhibited a sustained drug release over 4 weeks, although NPs in which the drug was adsorbed rather than entrapped exhibited a significantly higher biomineralisation and enhanced the expression of odontogenic genes and markers [117,141]. These were also tested on a LPS-treated dentine tissue model, confirming that the NPs promoted stem cell viability, migration, differentiation potential, and reduced inflammation, providing a suitable environment for tissue regeneration/repair [142].

It would be interesting to see how these systems perform in vivo, possibly even in a clinical pilot trial. Moreover, the physico-chemical and long-term stability and shelf-life of the various types of CS NPs should be evaluated, especially in the case of drug-loaded or chemically-conjugated NPs.

With a similar regenerative aim as Dasilva et al., Barreras et al. [31] confirmed that CS NPs could enhance the CHX antibacterial effect both on BHI–agar cultures and infected collagen membranes used for periapical tissue regeneration, although further studies are essential to establish the biological implications of using these nanomaterials in therapy. Furthermore, the effect of the treatment on the mechanical properties of the collagen membrane should be assessed.

**Figure 5:**
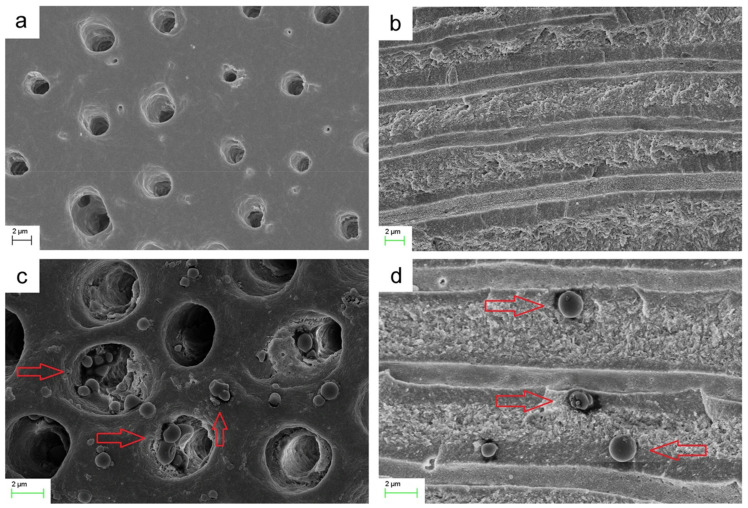
**Infiltration of PLGA NPs loaded with CHX, calcium, and phosphorous into dentinal tubules.** (**a**) Original dentinal tubule orifices; (**b**) original axial cross-section of dentinal tubules; (**c**) dentinal tubule orifices after NPs medication; (**d**) axial cross-sections of dentinal tubules after NPs medication. Red arrows indicate the NPs. Reproduced with permission from Fan et al. [52].

Fan et al. loaded CHX into PLGA NPs, together with calcium and phosphorus. This allowed the authors to achieve controlled release of the antimicrobial into dentinal tubules (with the auxilium of ultrasonic activation) (Figure 5) with a significant antibacterial effect, together with an increased dentin microhardness thanks to the inorganic loading, since dentin weakness is a common side effect of root canal medicaments. The NPs also exhibited excellent biocompatibility on murine osteoblasts [52].

Liu et al. investigated the possibility of replacing current endodontic strategies with tissue engineered dental pulp by repressing the inflammatory reaction and enhancing regeneration. The author challenged the LPS inflammatory response by transfecting dental pulp cells (DPC) with miR-146a (inhibitor mRNA for inflammatory mediatory signals), using PEG-PEI micelles as a vehicle, dispersed in an alginate gel also loaded with the fibroblast growth factor (bFGF). Results suggested that the gene delivery system significantly increased DPC proliferation 72 and 96 h after treatment in the presence of LPS. These preliminary results are quite promising, although there might be some stability issues related to the surface charge of the NPs as well as potential toxicity associated to high N/P ratios. These aspects would need to be thoroughly investigated before any potential clinical translation [79].

Toledano et al. evaluated their nanoMyP system for reducing radicular dentin permeability and facilitating dentin remineralisation, with the final aim to reinforce the root dentin and improve the fracture strength of the apical root after endodontic treatment. In particular, they assessed plain polymeric NPs and Zn NPs, Ca NPs, and Dox NPs, respectively. Dentin treated with Ca NPs and Zn NPs exhibited the lowest microleakage with hermetically sealed dentinal tubules and a zinc-based salt generation onto its surface. When using undoped NPs or Ca NPs, the deposition of minerals occurred, but they were imperfect and immature nanocrystallite structures, therefore radicular dentin was not mechanically reinforced. Root dentin treated with Dox NPs achieved the best organisation, quality, and collagen structure improvement, although they failed to exhibit sealing ability. The highest sealing efficacy was obtained in Zn NPs-treated samples, along with the highest values of Young’s modulus and dentin mineralisation. To proper translate these results into the clinic, the long-term effect of NPs should be evaluated, together with adequate in vivo assessments. Nonetheless, these NPs remain promising for their mechanical reinforcement and sealing ability of root dentin [85].

### 4.3. Periodontal Disease

The term “periodontal disease” includes a wide variety of chronic inflammatory conditions of the gingiva, bone, and ligament (the connective tissue collagen fibres that anchor a tooth to alveolar bone) supporting the teeth [143]. The pathogenesis of periodontitis involves a superinfection of dysbiotic periodontal biofilms [144] followed by an augmented immune response with the release of several inflammatory mediators such as interleukin (IL)-1, IL-6, IL-1β, and prostaglandins [145,146]. The vascular endothelial growth factor and the transforming growth factor-beta(1) have been also been shown to have significant effects on periodontal host response regulation [147]. Interestingly, recent studies have highlighted that a soluble urokinase-type plasminogen activator receptor, a marker of systemic immune activation, inflammation, and thrombogenesis could play an essential function in the development of coronary heart disease and periodontitis [148].

Clinically periodontal disease begins with gingivitis, the localised inflammation of the gingiva that is initiated by bacteria in the dental plaque. Chronic periodontitis occurs in susceptible individuals when untreated gingivitis progresses to the loss of the gingiva, bone, and ligament, which creates the deep periodontal “pockets” that are a hallmark of the disease and can eventually lead to tooth loss [143]. Genetic and environmental host factors influence the rate of the disease [149].

#### 4.3.1. Antimicrobial Activity

Current periodontal treatment requires conjunction of exceedingly painful mechanical cleaning with antibiotic administration. Systemic antibiotics require high doses, causing in turn unwanted side effects such as hypersensitivity, gastrointestinal intolerance, and development of bacterial resistance. Therefore, a more satisfactory approach would be to administer antimicrobial drugs directly into the pocket using a controlled release device. Local drug delivery limits the drug to its target site, with less or no systemic uptake. Unfortunately, localised delivery to the periodontal pocket is a tricky proposition as the flow rate of gingival crevicular fluid (GCF) perfusing from gums is greatly increased during periodontal infection. The site itself is rather active and is capable of dislodging a delivery device or washing away the released drug via salivary drainage [51]. Studies on the transport of NPs through the junctional epithelium revealed that NPs can provide a potential intra-pocket carrier system for the delivery of active substances to the periodontal pocket [49].

Aminu et al. in 2013 reported the preparation of triclosan-loaded PCL NPs using a quite elegant in silico Design of Experiment approach. The optimised formulation exhibited ~200 nm diameter, with remarkable entrapment efficiency and drug loading (>90 and >20%, respectively). The presence of the drug lowered the zeta potential from −7 to −34 mV, *de facto* increasing the colloidal stability of the NPs. Although all the payload was released within 3 h, therefore not highlighting any specific sustained or controlled release, NPs exhibited an outstanding shelf life of almost 18 months, even if this was limited to the chemical stability of the entrapped drug in the lyophilised samples [48]. It would have been interesting to reconstitute the lyophilised samples and assess also the size distribution and morphology of the NPs. Surprisingly, the author did not assess the antimicrobial properties of the NPs in vitro nor in vivo. Furthermore, a more apt cell type should have been chosen for biocompatibility assessment (*vide infra*).

In an effort to treat intracellular bacteria responsible for periodontitis (e.g., *P. Gingivalis*), also self-assembled block-copolymer vesicles (a.k.a., *polymersomes*) [13,150,151,152,153,154,155] were used to deliver metronidazole or Dox. Wayakanon et al. prepared polymersomes by self-assembly of amphiphilic poly [2-(methacryloy-loxy)ethyl phosphorylcholine] (PMPC) and poly [2-(di-isopropylamino)ethyl methacrylate] (PDPA) block co-polymers [156,157,158], reducing the number of intracellular *P. gingivalis* in patient-derived oral keratinocytes and fibroblasts, as well as organotypic cultures compared to unloaded polymersomes and free drugs used as controls [159].

To improve the drug treatment of paradontitis-causing biofilms, Takahashi et al. designed a formulation comprising an ionic liquid and CS NPs modified either with PLGA or with polyvinyl caprolactam—polyvinyl acetate—PEG graft copolymer (Soluplus^®^, Sol). The modifying polymer strongly affected the diameter of the NPs. When the NPs were incubated with bacterial biofilms, they increased the antibacterial ability of the ionic liquid, particularly in the case of Sol-modified NPs. Furthermore, the formulation of ionic liquid with the NPs improved its cytocompatibility, and the complete formulations showed enhanced retention capability even in the presence of simulated salivary flow. No in vivo assessment was performed, though, therefore limiting the translational capacity of this study [160].

Yao et al. prepared minocycline-loaded PEG-PLA NPs of around 100 nm in diameter. Most importantly, not only they confirmed the sustained in vitro release of minocycline (over 14 days), but they investigated the pharmacokinetics of minocycline in GCF in vivo. Minocycline concentration from NPs decreased slowly and retained an effective drug concentration for a longer time (12 days) than the control Periocline^®^ (i.e., minocycline formulated in Eudragit^®^ microparticles), with a significant reduction of periodontitis symptoms [69]. The same authors later introduced active targeting of the NPs by decorating their surface with an RGD peptide (which targets adhesion molecule integrin αvβ3), which significantly increased the NPs internalisation into epithelial cells, although no oral-specific cell types were used. The in vivo testing, however, confirmed an improved performance and sustained release in comparison to the non-targeted system [71]. Before clinical translation, though, it would be necessary to perform detailed studies on the specific antibacterial activity, as well as biocompatibility of the produced NPs with the gingival tissue during the residence time.

Singh et al. envisaged a mucoadhesive sodium carboxymethyl cellulose (SCMC) gel containing immunotherapeutic ganglioside coated polymeric NPs (G-PNPs) loaded with satranidazole (SZ). Usage of G-PNPs allowed to render SZ amorphous (i.e., more water dispersible than crystalline SZ). In vitro testing revealed qualitatively greater (~1.5-fold higher) antibacterial activity of the gel containing the NPs in comparison to the one loaded with crystalline SZ. Release studies highlighted an initial burst release followed by a more controlled kinetics, with about 70% of drug released in 24 h (an amount >MIC). Furthermore, immunomodulatory responses were observed in vitro in macrophage secreted cytokines (TGF-β, IL-4, IL- 12, TNF-α levels). Although the gel was also assessed in a clinical study (*vide infra*), it was not confirmed by the authors whether the immunomodulatory activity originated specifically from the ganglioside coating [51].

A similar system was developed by Beg et al., who exploited a Design of Experiments approach to develop an optimal in situ gelling drug delivery system (Poloxamer 407 + sodium chloride) bearing PLGA NPs loaded with moxifloxacin (MOX). The optimal drug delivery system exhibited a drug loading of 73%, which was released in a controlled manner over 7 days. The system was assessed in vivo in a rat periodontitis model, and compared to CHX and metronidazole as controls. An improved performance of the NPs-loaded in situ gelling system was observed within a week of the administration. Furthermore, radioimaging highlighted the high retention (>90%) and drug release of MOX in the periodontal pocket with high efficiency and specificity. It would have been interesting to assess the antimicrobial performance of this system, particularly on biofilms [161].

Aiming at an additional regenerative angle, Sindhura Reddy et al. developed injectable calcium-sulfate bone cement beads loaded with *O*-carboxymethyl CS tetracycline NPs (Tet NPs). The composite beads exhibited a sustained drug release pattern (with ~25% Tet released over 10 days), excellent antibacterial activity against *S. aureus* and *E. coli*, and biocompatibility for periodontal ligament cells. It would have been beneficial to assess this cement in vivo for its effect on the other indicators of periodontal disease [40].

With a similar purpose, Osorio et al. assessed their nanoMyP system loaded with calcium and zinc ions, which both exhibit antimicrobial activity [13,14]. Furthermore, calcium is associated with an improved osteogenic ability [11] and increased osteoconductivity [12], whilst zinc may also play a role in collagen protection from MMPs degradation [15]. After testing for 7 days in simulated bodily fluid, biomimetic precipitation of calcium phosphate deposits was observed, although in vitro testing on oral mucosa fibroblast cells showed a low dose-dependent cytotoxic effect, which should be further confirmed in vivo [17]. In a following study, the antibacterial effect was assessed on an in vitro subgingival biofilm model, which also included NPs loaded with silver and Dox. Reductions in bacterial viability were detected in biofilms in contact with the different NPs, more pronounced with silver and Dox NPs [82].

In the last years, as seen in the endodontal disease section, aPDT has also shown good promise for periodontitis treatment. Klepac-Ceraj et al. assessed the possibility of using aPDT to eradicate dental plaque bacteria causing chronic periodontitis in vitro using MB-loaded Pluronic^®^/PLGA NPs bearing a positive or negative surface charge. The NPs exhibited a marked difference in the release rate depending on the surface charge (>80% of encapsulated MB released from the cationic NPs in 12 h, compared to 28% from anionic NPs in the same timespan). The authors tested the NPs onto real patient-derived plaque samples of chronic periodontitis, both in suspension and following biofilm formation. However, although there appeared to be a reduction in bacterial viability, it was not significant in comparison to the controls. Further biological and microbiological characterisation of the interaction between the NPs and the bacterial, as well as mammalian cells should be undertaken, together with an optimisation of the aPDT parameters [119].

**Figure 6:**
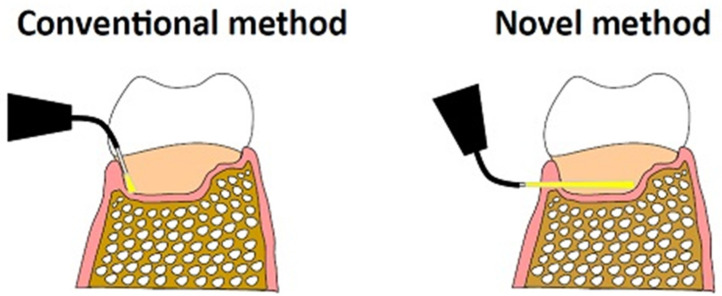
**Advantages of a novel irradiation method for extending aPDT into inaccessible areas.** On the left, the conventional irradiation method via the periodontal pocket (intra-pocket irradiation), where sufficient light cannot be delivered to inaccessible areas (e.g., furcation) due to the irradiation direction being parallel to the tooth axis. On the right, novel trans-gingival irradiation from outside the periodontal pocket (external irradiation), where light passing through the gingiva can penetrate deep into inaccessible areas. Modified with permission from Sasaki et al. [109].

Nagahara et al. investigated a similar aPDT approach, based on PLGA NPs coated with CS and loaded with indocyanine green (ICG) as a photosensitiser. Coating with CS ensured the attribution of mucoadhesive properties, and a significant bacterial reduction effect was achieved. Although the usage of ICG would allow to exploit high wavelengths (~800 nm), which can penetrate tissues more in depth, to avoid hyperthermia the treatment would have to be maintained below 60 s, therefore achieving a limited bactericidal effect [42]. In a follow-up study, the same authors further optimised their approach, evaluating it in vitro by delivering laser energy trans-gingivally (i.e., non-invasively) on a gingival model (3 mm beef slice) (Figure 6).

Results showed that the viability of a periodontal pathogen was significantly reduced by an order of 4 log_10_ by 5 min of laser irradiation, whilst the temperature increase was suppressed sufficiently with air cooling, to avoid thermal damage to periodontal tissues [109]. However, the authors did not confirm the generation of a singlet oxygen, and only used a single bacterial strain (*P. gingivalis*) in the planktonic phase, not biofilms. Moreover, the effects of ICG NPs on host cells were not evaluated. Further studies are needed to reveal the mechanisms of bacterial inactivation (including the possibility of a thermal effect) and to ensure the safety of this method for clinical application.

#### 4.3.2. Bone Regeneration/Resorption Inhibition and Immunomodulation

Periodontal disease is characterised by chronic inflammation in the tissues that protect and support the tooth. The use of anti-inflammatory agents may contribute to the modulation of the host response, reducing the severity of inflammation, connective periodontal tissue degradation, and bone loss. Based on this hypothesis, Napimoga et al. prepared PLGA NPs loaded with 15d-PGJ2 and assessed their immunomodulatory effects in a mouse periodontitis model. Infected animals treated with the 15d-PGJ2 NPs presented significantly lower bone resorption than infected animals without the treatment. The usage of NPs as a delivery system achieved a >30-fold reduction of the drug dosage [162]. A marked reduction of inflammatory cells was observed in the submandibular lymph node, especially at the highest dose (10 mg/kg). Expression levels of the osteoclastogenesis-inducing factor RANKL and several proinflammatory cytokines and chemokines were decreased, whereas CD55, an antiadhesive molecule that promotes the clearance of epithelial-bound neutrophils, was elevated. The study, however, could have benefitted from a more thorough physico-chemical characterisation of the NPs (e.g., size, colloidal stability), as well as a more thorough biodistribution assessment of the administered NPs [163].

Ma et al. confirmed that the recruitment of macrophages at the inflamed periodontal region can actually be exploited to deliver CS NPs after intraperitoneal administration. Although the authors have loaded siRNA into the NPs, achieving a selective accumulation at the inflamed site by >2-fold, it would be potentially possible to translate this strategy also to other drug payloads [164].

PLGA/CS NPs loaded with lovastatin and Tet were prepared and assessed for chronic periodontitis potential treatment. Whilst Tet elicits an antibacterial action, secondary effects of statins include anti-inflammatory and antioxidant properties, and stimulation of bone formation by increasing the expression of the BMP-2 gene in bone cells [165,166]. The NPs exhibited different release profiles for the two drugs (slower for lovastatin, marked burst effect for Tet), with good biocompatibility and bactericidal activity. Furthermore, a significant new bone formation was observed (25% more) in the treatment group when compared to the control, when the NPs were administered in a canine periodontitis animal model [54]. The preparation of the NPs (via double emulsion), however, is poorly suitable for potential large-scale applications. Moreover, the NPs dispersed in gelatin (used for the in vivo experiments) would have benefitted from a dedicated characterisation.

Pramod’s group prepared and tested eugenol-loaded PCL NPs of ~200 nm and negative surface charge (~ −30 mV), which ensured stability against aggregation. The drug load was almost completely released within the first 12 h, and albeit the in vitro assessment should be improved (*vide infra*), in vivo experiments in a periodontitis rat model indicated that eugenol-loaded NPs could prevent septal bone resorption, whilst the free drug did not elicit any response [49].

With the aim of exploiting mitogen-activated protein kinase phosphatase (MKP)-1 expression to regulate periodontal inflammation, Valerio et al. prepared and tested in vitro and in vivo PEG-PLA NPs loaded with auranofin (ARN), an antirheumatic drug that induces MKP-1 expression. Data indicated that ARN-NPs had reduced cytotoxicity compared with free ARN, with an induction of Dusp1 mRNA expression (which codifies for MKP-1). Flow cytometry indicated that NPs were rapidly uptaken into macrophages (within 2 h). Finally, a significant bone loss reduction was observed with ARN-NPs compared with control NPs in vivo using a periodontitis rat model [107].

Although promising, a more detailed evaluation of the biological effect on the inflammation profile (as well as antibacterial activity in the case of Pramod’s study) of the NPs would have been desirable.

NPs formulations could also actively promote bone regeneration and new osteodeposition. For example, Xue et al. produced CS, PLGA, and silver NPs for periodontal tissue regeneration. A ratio of 50 µg/mL of silver NPs mixed with PLGA and CS NPs (7:3) resulted in an increase in bone density and healing in vivo (in rabbits) with respect to controls, although the antimicrobial activity would have to be optimised, perhaps via the introduction of a suitable drug load [167].

An interesting approach has been investigated by Capretto et al., who prepared Pluronic^®^ polymeric micelles loaded with dexamethasone and ascorbic acid 6-palmitate to drive the osteogenic differentiation of human periodontal ligament mesenchymal stem cells (hPDLSCs). A microfluidic approach was exploited to produce monodisperse and regular micelles, which successfully differentiated hPDLSCs in their osteogenic lineage. Although the microfluidic system was characterised and optimised in depth, the molecular biology of the osteogenic differentiation event would need to be elucidated more in detail for future potential clinical applications [66].

Aiming at achieving a sustained drug release, Mazzarino et al. have developed mucoadhesive CS-coated PCL NPs loaded with curcumin, and further dispersed them into a CS film to create a mucoadhesive drug dosage form. Authors tested CS with different MW, highlighting a direct proportion between CS MW, diameter (up to 250 nm), and drug loading of the NPs. Films, however, could not be prepared using low-MW CS due to the high heterogeneity obtained, but the presence of NPs did not affect the properties of the obtained films. The swelling properties evaluated were in line with the obtainment of suitable mucoadhesive properties, with an extremely controlled drug release (~5% over 24 h). It would be interesting to assess the delivery over longer periods, therefore verifying for how long such a system can indeed be used. Furthermore, an assessment of the system in vitro and in vivo should be carried out [43].

### 4.4. Oral Cancer

Oral cancer is the 16th most common malignancy in the world [168]. The common approaches to oral cancer treatment can be divided into: surgery, radiotherapy, and chemotherapy. However, the collateral effects induced by these techniques have oriented the scientific community to investigate alternative solutions based on nanotechnology.

The group of Krishnakumar evaluated the possibility of using naringenin (NAR, a flavonoid naturally present in citrus fruits) delivered in Eudragit^®^ NPs to prevent the formation of OSCC. With an encapsulation efficiency of ~90%, the oral administration of NAR NPs for 16 weeks in hamster OSCC-models completely prevented tumour formation. Furthermore, the authors cleverly exploited FT-IR and Raman spectroscopy to evaluate the carcinogenesis process (or its drug-loaded NPs-mediated inhibition) at a molecular level (in terms of changes in the amounts and order of lipids, sugars, proteins, and nucleic acids) [91,92,93,97]. With a more therapeutic approach in mind, the same group also evaluated the possibility of delivering poorly water-soluble hesperetin [98,99] and silibinin [95] with the same NPs formulation. Both are plant derived flavonoids, isolated from citrus fruits (hesperitin) and fruits and seeds of the milk thistle (silibinin). Silibinin, in particular, exhibits G1 arrest and apoptosis effects (although this was erroneously evaluated by the authors on non-oral cancerous cell lines, *vide infra*), which are useful adjuvants for chemotherapy [95]. These NPs also exhibited a partial tumour prevention effect (albeit lower than NAR NPs), and they improved the status of endogenous fluorophore emission (as evaluated via the analysis of autofluorescence spectral signatures) to a near normal range in hamster OSCC-models, which are characterised by a decrease in the autofluorescence emission [96,99], as well as by an increase in endogenous porphyrins emissions, possibly connected to hypervascularity [98] in comparison to the control tissues.

Mariadoss et al. loaded a different plant-based compound, phloretin, into CS NPs. NPs exhibited a pH-dependent release behaviour (at physiological pH ~20% the drug was released after 48 h, whilst in acidic pH mimicking the tumoural environment this value increased to >70%). The overall IC_50_ value calculated for NPs was not too dissimilar from the one of the free phloretin, therefore questioning the need for NPs as drug delivery systems. Furthermore, the authors should have selected more carefully the cell types for the in vitro assessment, not only the cancerous ones (*vide infra*), but also the non-cancerous lines, since these should reflect the healthy surrounding oral tissues. Moreover, no in vivo assessment of the NPs was performed [169].

Endo et al. exploited a micellar preparation of PEG-poly(glutamic acid) loaded with cisplatin (labelled NC-6004) to obviate the side effects related to this drug (namely nephro- and neurotoxicity) when administered systemically. Although the differences in tumour inhibition effect between the encapsulated and free drug were not significant, the level of toxicity was dramatically reduced (66% less) when cisplatin was vehiculated via the micelles. Furthermore, sentinel lymphnodes could be targeted via the micellar system, whilst the free drug did not enter the lymphatic system [63].

**Figure 7:**
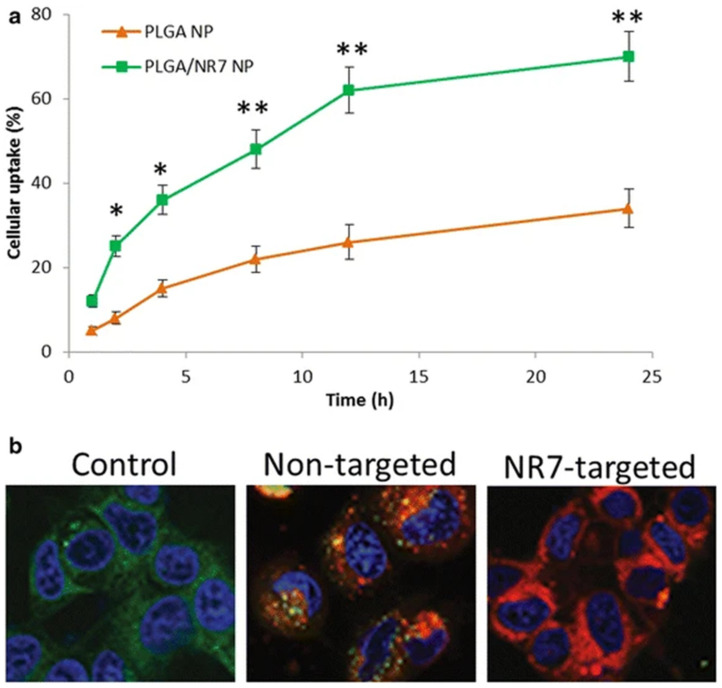
(**a**) Intracellular uptake of PLGA NP and PLGA/NR7 NPs in HN6 squamous cell carcinoma. Rhodamine B was used as a fluorescent dye. The uptake is shown as a percentage of total amounts of NPs (dye) incubated with the cancer cells. (**b**) Representative confocal microscopy images of targeted and non-targeted NP in HN6 cancer cells. The cells are stained with the Lysotracker lysosomal stain and DAPI was used to stain the nucleus. ** *p* < 0.01 and * *p* < 0.05 are the statistical differences between the cellular uptake of two formulations. Reproduced with permission from Wang et al. [64].

Rather than poly(glutamic acid), Wang et al. used PLGA as the hydrophobic portion to produce PEG-PLGA polymeric micelles. Furthermore, they conjugated an epidermal growth factor (EGF) receptor-targeting motif on the surface of the NPs, since EGFR is highly expressed in human epithelial cancer cells such as OSCC. The drug was released in a controlled manner, without any type of burst release over 96 h. The targeting moiety allowed increasing the internalisation in target cancer cells by 2-fold over 24 h, exacerbating the importance of a selective targeting moiety for drug delivery. This behaviour was also reflected in the cytotoxicity studies, which the authors confirmed were being attributed to apoptotis (Figure 7) [64].

In the case of cisplatin-resistant cancer cells, curcumin (which also exhibits an anticancer potential) has been loaded onto PLGA NPs and has been found to induce cell apoptosis starting from concentrations as low as 20 µM, with little or no cytotoxicity on healthy gingival fibroblasts and oral keratinocytes. The authors of the study, however, did not elucidate in much detail the drug loading and release properties of the NPs in comparison to the free drug, which would have strengthened the importance of their choice for using NPs as a delivery system [170].

**Figure 8:**
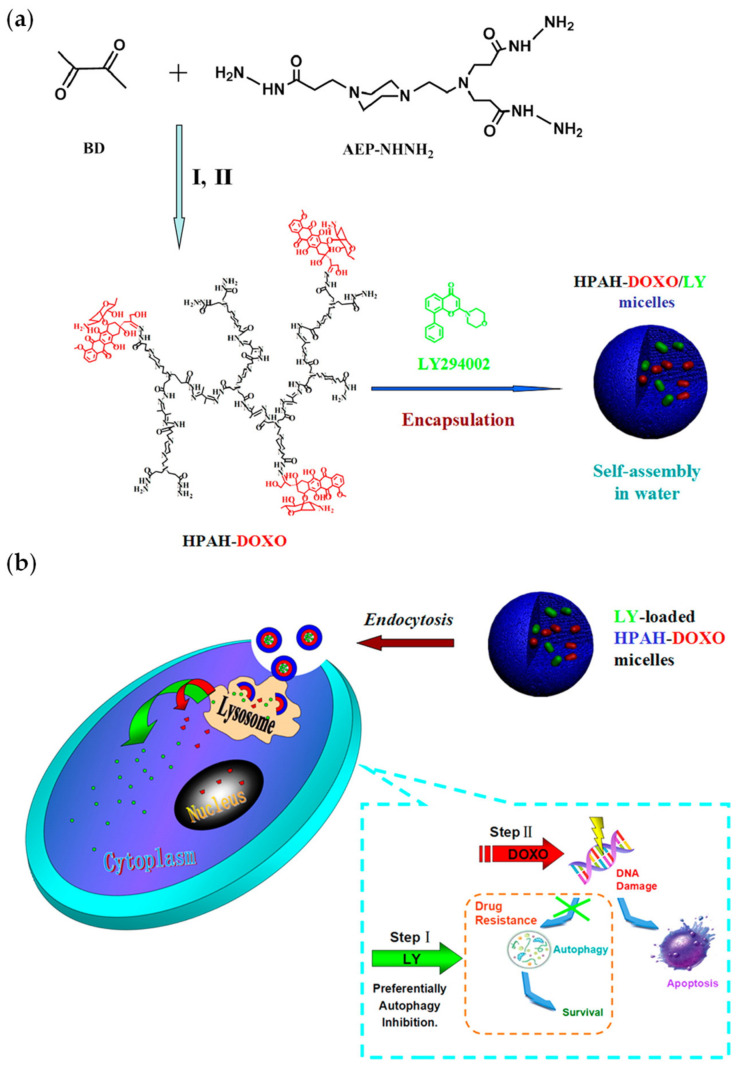
(**a**) Synthesis route of HPAH−DOXO and construction of the LY-loaded HPAH−DOXO micellar NPs. I, HPAH was synthesised via polycondensation of BD and AEP-NHNH2; II, conjugation reaction between DOXO and HPAH to obtain HPAH−DOXO. (**b**) Proposed mechanism of cellular uptake of the LY-loaded HPAH−DOXO micellar NPs and intracellular drug release. Modified with permission from Saiyin et al. [62].

Saiyin et al. developed an effective chemotherapeutic system for OSCC based on polymeric micelles for codelivery of the anticancer drug doxorubicin (DOXO) and the autophagy inhibitor LY294002 (LY). DOXO was covalently conjugated onto pH-responsive hyperbranched HPAH, therefore resulting in the formation of an amphiphilic derivative which was self-assembled into reasonably monodisperse micelles of ~40 nm, capable of loading the poorly water-soluble and relatively toxic LY. The release of DOXO and LY was pH-dependent, with LY being released significantly faster than DOXO at mild acidic pH (pH = 5) (Figure 8). In vitro studies demonstrated that the LY-loaded HPAH−DOXO micelles could enter cancer cells within 1 h and then release LY and DOXO in response to an intracellular acidic environment. Compared to the HPAH−DOXO micelles and the physical mixture of HPAH−DOXO and LY, the LY-loaded HPAH−DOXO micelles induced a higher (2- to 3-fold) proliferation inhibition of tumour cells, illustrating a synergistic effect of LY and DOXO over 48 h. The authors hypothesised that the preferential release of LY inhibited the autophagy of tumour cells and made them more sensitive to the subsequent liberation of the conjugated DOXO [62].

However, no in vivo assessment of the formulation was undertaken for these above-mentioned studies, therefore limiting the translational potential of the produced NPs.

A similar approach was used by Shi et al. by conjugating docetaxel to PEG-PLA copolymers to produce micelles [74]. This has allowed achieving an extensive sustained release over 160 h, moderately accelerated by an acidic environment. Although the free drug was more potent within the first 24 h when tested in vitro, in 48 h the drug delivery system resulted in a higher anticancer activity. When tested in vivo, the tumour growth was 3-fold lower in mice treated with the micelles in comparison to the free drug. It would be certainly interesting to evaluate the performance of these systems into an orthotopic model rather than an ectopic one.

Li et al. maximised the chemical versatility offered by polymeric NPs to integrate stimuli-responsiveness, as well as active targeting into PEG-PLGA micelles. They did so by introducing a thioketal-containing linker responsive to ROS (which are overproduced in tumour cells when compared to healthy cells) between the PEG and PLGA portion of the copolymer, and decorating the PEG extremity with an RGD-peptide. The authors loaded DOXO into the micelles, achieving a release behaviour dependent from the concentration of oxidative species, successfully evaluated both in vitro and in vivo. To maximise the DOXO release, however, the authors had to co-load a drug (alpha-TOS) to further upregulate the ROS production. Furthermore, also in this case the animal model chosen was a xenograft ectopic one [70].

An innovative approach towards the treatment of OSCC has been developed by Lin et al., who elegantly produced Cu(II)-loaded CS NPs to be employed in photothermal therapy (i.e., the non-invasive ablation of malignant tissue by heating the tissue locally above 43 °C on the basis of the photothermal effect of NPs) combined with the chemotherapeutic effect via the release of Cu(II). After loading cupreous complexes into CS and forming the NPs, a photothermal therapeutic effect was observed on KB cells in vitro and KB tumours in vivo. Moreover, the post-treatment toxicological parameters were evaluated, highlighting the absence of long-term toxicity [38]. The authors, however, should have chosen a more suitable model for the assessment in vitro and in vivo (*vide infra*).

Pornpitchanarong et al. investigated the possibility of improved mucoadhesiveness by modifying DOXO-loaded CS/hyaluronic acid NPs using catechol. Their hypothesis was that the addition of catechol would have allowed increasing the residence time of NPs into the oral mucosa thanks to the establishment of chemical interactions with mucin proteins. Although only a limited improvement of the mucoadhesive properties was observed in an ex vivo model of oral mucosa, this was indeed statistically significant. Surely with further optimisation (also of the drug release and loading) such a strategy could be employed more widely [171].

## 5. From Bench to Clinic. The “Bench” Side: Testing the NPs for Oral Diseases

### 5.1. Assessing Drug Release from NPs

Undoubtedly, one characteristic that makes NPs so interesting for biomedical applications is the possibility of predicting and or controlling the release of a payload. Despite this great tunability, it is also crucial to consider that the external factor inducing the NPs disassembly/degradation and subsequent payload release should fall in the pathophysiological ranges of the target tissue. The medium present in the mouth is saliva. This is a clear, mucinous-serous secretion, comprised of different electrolytes, proteins, peptides, polynucleotides, and organic molecules. Most importantly, saliva also contains enzymes and bactericidal substances such as lysozyme, lactoferrin, and salivary peroxidase, and its pH (6.8–7.2 in physiological conditions) can range between 5.7 and 8 according to the food ingested and the presence of pathological conditions [172]. Taking this into consideration, it seems reasonable to think that NPs stability studies and assessments of controlled release should be performed in fluids that closely resemble the salivary composition rather than bacterial broths or simple aqueous buffers. The use of simulated saliva of various compositions was reported. Examples include artificial saliva (pH 7.4) containing 0.6% (*v*/*v*) Tween 80 by Tiyaboonchai et al. [76], simulated saliva (pH 6.8) with bovine serum albumin/PBS (1/4, *v*/*v*) by Chronopoulou et al. [30] or saliva from commercial sources [136]. Indeed, the availability of standardised saliva substitutes or other oral fluids (e.g., subgingival or GCF), could be the solution for a more robust and uniform testing of NPs.

### 5.2. Determining the Antimicrobial Effects

NPs are usually tested using disparate biofilm growth conditions combined with various approaches to examine biofilm viability. This type of assessment is carried out on a single bacterial species (often *S. mutans*) [24,44] or on multispecies [128]. This lack of consistency, often given by the complexity of the tissue, renders the comparison of efficacy among treatments and studies extremely complicated. Moreover, despite the technical and analytical challenges related to the use of polymicrobials, the use of single species for testing the therapeutic efficacy of a treatment is reductive and provides a limited overview of the possible effects.

In a recent review, Kreth et al. comprehensively summarised the weaknesses and strengths in the approaches used to assess the interactions between biomaterials and biofilms. The authors also provided a series of suggestions on how to standardise the testing conditions across studies [173]. All the advices proposed by Kreth et al. are perfectly valid also for the world of NPs and should represent the gold standard to perform robust testing. Nonetheless, it is important to remember that assessing the multifactorial dynamics of interactions in biology—particularly the connections between pathogens-commensal microbiota and host-cells—is extremely challenging.

Recreating these dynamics in vitro would require a multidisciplinary effort supported by state-of-the-art techniques that are often difficult to access. Therefore, there is the need for different approaches. Paraphrasing a famous sentence from the “The Art of War” by Sun Tzu, “…*know your bacteria and you need not fear...*”, a personalised medicine approach could perhaps be the solution. Multispecies biofilm stocks derived from plaque sampling [88,119] could be characterised using high-throughput screening to determine the microbiota exact composition and abundance, and used to test the efficacy of a therapy. Arguably, this would be the most representative form of oral microbiota that could be employed for testing microbial interactions. An important aspect to be considered when the therapy aims to challenge the oral bacteria is the effect exerted by the payload on the surrounding host tissues and the commensal microbiota. As previously described, the oral microbiota has a central role in the homeostasis of the tissue. Targeting the pathogens with antimicrobial molecules that also affect the commensals could not only be therapeutically inefficient, but also exacerbate the situation leading to worst repercussions. Hence, it is fundamental to assess the “big picture” when dealing with tissue-dynamics.

### 5.3. In Vitro Models

Alongside using the most representative model for the oral microbial community, it is equally critical to employ the correct host-cellular models. Among the studies described in this review there is a wide range of in vitro models that were used. For example, tooth regeneration following delivery of osteogenic molecules has been correctly assessed on human mesenchymal stem cells derived from periodontal ligaments [66] or on primary cultures of human bone marrow-derived osteoblasts (HOB) [54]. Another interesting model is represented by the stem cells from the apical papilla (SCAPs). This is a population of mesenchymal stem cells (MSCs) residing in the apical papilla of immature permanent teeth and presenting self-renewal and differentiation capabilities [174]. SCAPs are finding wide application in the field of oral care and are a robust model for testing regeneration of dental tissues [117,141]. The use of human derived stem cells is clearly preferable to cell lines, although the ethical restrictions linked to the use of these cells could be challenging. Therefore, alternatives such as MC3T3-E1 cells, a cell line derived from the calvaria of newborn mice, represent a good alternative for studying in vitro osteoblast differentiation and activity [52].

Regarding the investigations of polymeric NPs for treating oral cancer, the in vitro models generally used are represented by squamous carcinoma cells. This carcinoma is the most common malignant neoplasm found in the oral cavity [175]. From this multifaceted cancer a variety of cell lines have been derived that have been employed in various studies. Examples are HSC3 cells [74] and Cal27 [62,70] from human tongue squamous carcinoma or HN6 cells [64] from head and neck squamous carcinoma. Unfortunately, there are also examples of misused models. Different studies, that were focusing on the oral application of polymeric NPs, employed L929 cells as a model [48,49]. This widely used cell line is, however, derived from mouse subcutaneous adipose connective tissue, and therefore poorly represent the malignancies developing in the oral tract. Importantly, another type of cell that is often used to model oral cancer and that is referred to as KB cells [38,95,169], allegedly a squamous cells carcinoma, are actually HeLa cells (cervical carcinoma cell line) [176]. In fact, the KB cell line allegedly established in 1955 by Harry Eagle [177], was demonstrated to be misidentified by Stanley Michael Gartler in 1967 [178] and also the original stocks of KB deposited at the American Tissue Culture Collection were demonstrated to be HeLa [179]. Although in vitro models are clearly a simplistic representation of the true nature of a tissue or disease, they are the solid basis for determining the therapeutic effect of novel treatments. Therefore, it is absolutely critical that such models are not only relevant to the purpose of the investigation, but also the correct ones.

### 5.4. Ex Vivo Models

One methodological approach to increase the degree of complexity of an in vitro model is the use of ex vivo systems or 3D matrices that resemble the native tissue environment. In the field of oral disease, dentin is by far the most used model. Dentin is a calcified tissue comprised of organic minerals (70% of which is hydroxyapatite), extracellular matrix (ECM, mainly collagen type I), and cells [180]. Dentin can be easily sourced by animals or humans and it provides the optimal substrate for assessing not only the interaction of cellular components (bacteria or stem cells) with the ECM, but also to determine the effect of therapeutic treatments.

**Figure 9:**
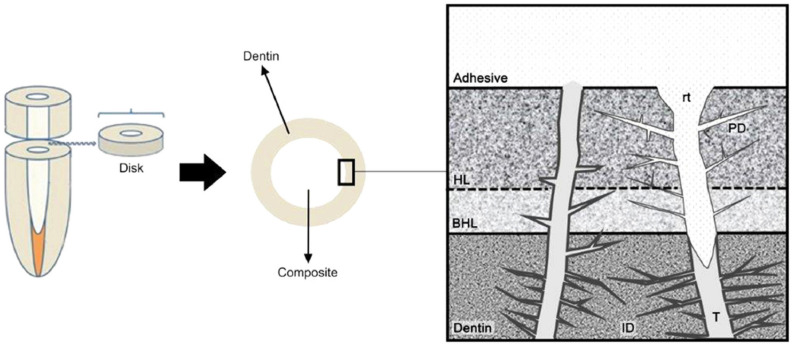
Schematic illustration presenting disks preparation mode and the observation zone (the resin-dentin bonded interface). HL: hybrid layer; BHL: bottom of hybrid layer; PD: peritubular dentin; ID: intertubular dentin; rt: resin tag; T: dentinal tubule. Reproduced with permission from Toledano-Osorio et al. [88].

Recently Toledano-Osorio et al. described the derivatisation of dentin disks from bovine incisors to assess the effects of their nanoMyP system to protect the resin-dentin interface from cariogenic biofilm (Figure 9) [88].

The same group also detailed the protocol to create dentin blocks from the buccal surface of single-rooted teeth obtained from young donors [84]. Similarly, Genari et al. obtained dentin disks from healthy upper premolar teeth to test [94], while Priyadarshini et al. used non-carious and non-restored human molars for the same purpose [50]. In a different work, the same lead author used an ingenious approach to simulate native conditions. To mimic the positive hydrostatic pulpal pressure, a custom-built device was connected to the fluid-filled pulp chamber containing dentin specimens anchored to a glass support. Using this strategy, it was possible to assess the efficiency and depth of drug delivery in the tissue against physical forces that are inherently present within the target tissue [50]. Although the setup and optimisation of these custom-built devices is not trivial (as observed for the recurrent discrepancies in drug release profiles), the surge of “on a chip” models able to resemble the native environment [181] could ease the transition to more complicated, reliable, and pertinent ex vivo systems.

### 5.5. In Vivo Models

The final test, and perhaps the most important, of any biological assessment is in vivo. Despite all the efforts invested, we are far from being able to recapitulate the real pathophysiological conditions in vitro. Therefore, the use of in vivo models is still required. Clearly the type of model used is dictated by the goal of the study.

An important in vivo model used for assessing periodontal inflammation and regeneration is the ligature-induced periodontitis, where the cervical region of the teeth is tied using dental ligature wires. This procedure is thought to facilitate the accumulation of microbiota enhancing the inflammatory state [182], and it is widely used in large animals. For example, it has been used in beagle dogs to assess the efficacy of minocycline-loaded NPs for attenuating inflammation [69,71], but it has been adopted also in rats to test eugenol-loaded NPs against periodontal infections [49]. A different way to induce inflammation and consequent tissue damage is the direct injection of inflammatory molecules. Examples are the use of *A. actinomycetemcomitans* in rats or mice [107,163] or LPS of *P. gingivalis* in mice [164]. The same approach was also proposed by Sakima et al., who inoculated mice with *C. albicans* to test antifungal PDT mediated by curcumin-loaded NPs [108]. However, it must be highlighted that the authors reported the death of 13 animals before the end of the experiments, six of which during *C. albicans* inoculation, casting ethical concerns regarding the validity of the model. Finally, the xenograft transplantation of human cancer cells in animal models, which is the gold-standard used in research and testing of anti-cancer therapies, was also employed in the field of oral cancer. One example reported in this review is the administration of the oral tongue Cal27 cell line in the back of BALB/c mice [70]. Although the testing on human cancer cells is important, the successful growth of such cells in vivo requires the use of immunocompromised animal models. This is a limitation, particularly when assessing the efficacy of NPs as a delivery system. In fact, upon systemic administration, NPs interact with various components of the immune system which might hinder their efficacy [183]. Therefore, in order to have a more reliable assessment of the therapeutic efficacy of these drug delivery systems it is advisable to employ fully immunocompetent models. In this regard, a valid alternative is represented by the hamster buccal pouch carcinoma induced by 9,10-dimethyl-1,2-benzanthracene (DMBA). This model was employed to test hesperetin loaded NPs [97,98]. Nonetheless, it should be underlined that also this model is not free from limitations. The human oral cavity lacks the buccal pouch where the tumour is induced, and such pouch possesses a mucosal tissue that is histologically different from the human one. Furthermore, the etiological and genetical composition of this tumour is significantly different from the human oral carcinomas [184].

All considered, no animal model perfectly recapitulates human pathophysiology. However, the general consensus it that using suitable preclinical models is helpful, it allows for an in-depth investigation of the biological effects of a given therapy and it can help bridge the gap between bench research and clinical translation.

## 6. From Bench to Clinic. The “Clinic” Side: Clinical Trials of NPs for Oral Diseases

Although it is still in its early stages, the clinical investigation of polymeric NPs in the treatment of oral conditions is advancing.

**Figure 10:**
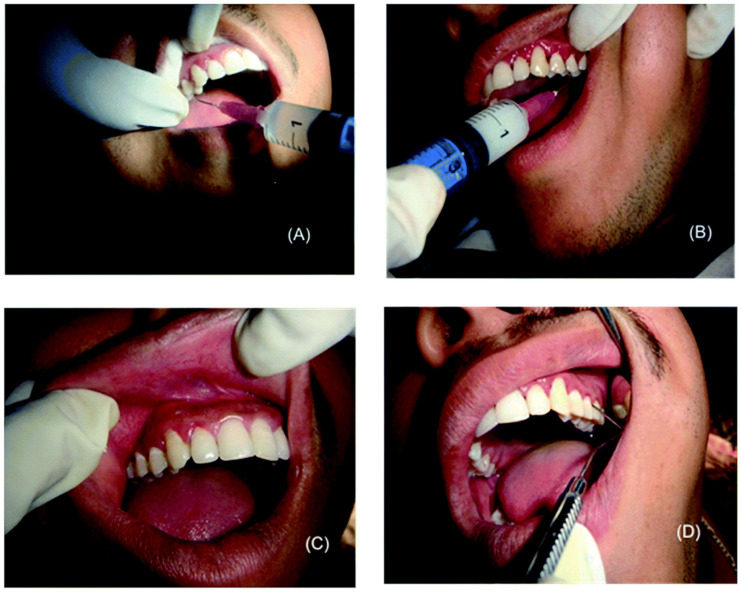
Snapshots of a patient undergoing a clinical study detailed in Singh et al. The trial was split mouth, i.e., locations for groups designated as A and B were both situated in the mouth of the same person. (**A**) Gel containing conventional SZ and (**B**) SCMC gel bearing G-PNPs loaded with SZ being administered on the 0th day; (**C**) and (**D**) show level of healing on the 21^st^ day in groups A and B, respectively. The indentations have filled up, with no evidence of bleeding on probing. Reproduced with permission from Singh et al. [51].

For example, there are encouraging preliminary clinical data of using NPs for the treatment of periodontitis. The SCMC gel bearing G-PNPs loaded with amorphous SZ developed by Singh et al. [51] has been evaluated in a 21-day single blind clinical trial in comparison to the same gel containing conventional crystalline SZ (Figure 10).

Parameters of the comparison included texture, mucoadhesion, drug release, and inhibitory susceptibility of *A.* *actinomycetemcomitans*. The gel bearing the NPs caused a significant (*p* < 0.05) decrease in clinical markers of periodontitis, i.e., gingival index and pocket depth as compared to the conventional gel. Moreover, a reduction in the plaque index produced was highly significant (*p* < 0.01) at the end of the 21st day of clinical study.

In addition to the in vitro assessment on planktonic and biofilm phases, De Freitas et al. investigated MB-loaded PLGA NPs in a clinical pilot study with 10 adult human subjects with chronic periodontitis. Patients were treated either with ultrasonic scaling and scaling and root planning (US + SRP) or US + SRP + aPDT with MB-loaded NPs in a split-mouth design. The clinical study demonstrated the safety of aPDT, and although both groups showed similar improvements of clinical parameters after 1 month from the treatment, at 3 months US + SRP + aPDT showed a greater effect (~30%) on gingival bleeding index compared to the group treated solely with US + SRP, whilst the other parameters all returned to baseline levels, possibly due to bacterial recolonisation which is peculiar of chronic periodontitis. For adequate application, the appropriate aPDT dosimetry (photosentisiser concentration, incubation time, power density, and energy fluence) will have to be defined to effectively eradicate biofilm species [56].

A single local application of curcumin-loaded PLGA/PLA NPs as an adjunct to SRP in nonsurgical periodontal treatment was tested in a clinical study on 20 subjects with periodontitis. Participants received PLGA/PLA NPs loaded with 50 μg of curcumin or empty NPs. Probing pocket depth, clinical attachment level, and bleeding on probing were monitored at baseline, 30, 90, and 180 days, in addition to IL-1α, IL-6, TNFα, and IL-10 in the GCF being assessed by ELISA. Unfortunately, this study showed no significant differences between the two experimental groups, with no additional benefit for the groups receiving the single local administration of curcumin-loaded NPs [185]. On the other hand, a recent trial was conducted by Guru et al. where the efficacy of 2% curcumin-loaded Pluronic^®^ nanogels and conventional 1% CHX gel was compared in the treatment of periodontal pockets. The study included 45 patients with chronic periodontitis who received SRP followed by random allocation to the three treatment groups, namely no further intervention (Group 1), 2% curcumin nanogels (Group 2), and 1% CHX gel (Group 3). A clinical parameter assessment and microbiological analysis of subgingival plaque samples for *A. actinomycetemcomitans, P. gingivalis,* and *T. forsythia* was done at baseline, 21^st^ day, and 45th day. Both experimental groups exhibited a comparable antibacterial effect on the three selected periodontopathic bacteria with similar improved clinical parameters [186]. This highlights the importance of the formulation to generate the NPs even in the presence of the same drug.

Nanotechnology based therapy approaches have also attracted great interest in oncology in recent years [187]. Although the majority of clinical trials are based on liposomal NPs or albumin-conjugates [188,189,190,191], NC-6004, the micellar preparation of PEG-poly(glutamic acid) loaded with cisplatin developed by the group of Kataoka [63] is under clinical evaluation. A recent open-label, nonrandomised, phase Ib/II trial showed the tolerability and promising activity of NC-6004 in combination with gemcitabine [192]. In this study, 22 patients with advanced solid tumours, including two patients with squamous cell head and neck cancer, received NC-6004 at 60 to 180 mg/m^2^ on day 1 and gemcitabine at 1250 mg/m^2^ on days 1 and 8 every 3 weeks. Although the results were not stratified for the type of cancers, the most common grade III/IV hematologic adverse events were leukopenia (68%) and thrombocytopenia (59%) with tumor shrinkage occurring in 55% of participants, partial responses in 15%, and stable disease in 70%. Most patients reported stable or improved quality of life score, as assessed by the EORTC QLQ-C30 questionnaire.

A Phase IIa/IIb clinical trial of NC-6004 plus pembrolizumab in patients with head and neck cancer who have failed platinum or a platinum-containing regimen (NCT03771820) is ongoing and estimated to be completed by 2022 [193]. The aim of the Phase IIa of this trial is to determine the optimal NC-6004 dose in combination with pembrolizumab with the aim of the Phase IIb being a head-to-head comparison in the progression-free survival of NC-6004 plus pembrolizumab vs. pembrolizumab alone.

## 7. Future Outlook and Perspective

This comprehensive review has shown that nanotechnology and NPs hold a considerable potential to improve the prevention and treatment of oral diseases, representing an extremely attractive topic for future investigations. We have highlighted the most popular polymeric materials which are currently used to generate NPs aimed at oral health applications, as well as the production and cargo-loading methods. We have illustrated a plethora of applications, thoroughly explored in some cases all the way to the clinical assessment. Furthermore, we have highlighted the advantages, disadvantages, and challenges of the in vitro, ex vivo, and in vivo models currently used for NPs characterisation. Although most approaches are still in the preclinical stages, they have shown tremendous potential to fulfill the need for viable alternative preventative and therapeutic strategies. Nonetheless, based on the limited number of clinical trials performed thus far, it is evident that there is still a long road ahead to properly achieve the ultimate goal of personalised medicine. To the best of our knowledge, currently there are no commercially available products based on NPs technology. To pursue this, it is certainly vital to shift the usage of non-biodegradable acrylates and methacrylates to FDA-approved or generally recognised as safe (GRAS) materials. Undoubtedly, there might be some challenges related to a reliable scale up for the reproducible production of the polymeric NPs. Nonetheless, judging by the growing number of patents and papers over these past years, as well as by the enhanced possibility of local rather than systemic administration of these nanosystems, we are confident that the development of the first commercial polymeric NPs products against oral diseases is not far.
